# Epidemiological Surveillance, Variability, and Evolution of Isolates Belonging to the Spanish Clone of the 4,[5],12:i:- Monophasic Variant of *Salmonella enterica* Serovar Typhimurium

**DOI:** 10.3390/antibiotics14070711

**Published:** 2025-07-16

**Authors:** Xenia Vázquez, Patricia García, Javier Fernández, Víctor Ladero, Carlos Rodríguez-Lucas, Jürgen J. Heinisch, Rosaura Rodicio, M. Rosario Rodicio

**Affiliations:** 1 Departamento de Biología Funcional, Área de Microbiología, Universidad de Oviedo (UO), 33006 Oviedo, Spain; fernandezdjavier@uniovi.es; 2 Grupo de Microbiología Traslacional, Instituto de Investigación Sanitaria del Principado de Asturias (ISPA), 33011 Oviedo, Spain; carlos.rodriguezlu@sespa.es (C.R.-L.); mrosaura@uniovi.es (R.R.); 3 Department of Microbiology, Biomedical Research Institute A Coruña (INIBIC), University Hospital A Coruña (CHUAC), 15006 A Coruña, Spain; patricia.garcia.fernandez@sergas.es; 4 Centro de Investigación Biomédica en Red de Enfermedades Infecciosas (CIBERINFECT), Instituto de Salud Carlos III, 28029 Madrid, Spain; 5 Servicio de Microbiología, Hospital Universitario Central de Asturias (HUCA), 33011 Oviedo, Spain; 6 Centro de Investigación Biomédica en Red-Enfermedades Respiratorias, 30627 Madrid, Spain; 7 Research & Innovation, Artificial Intelligence and Statistical Department, Pragmatech AI Solutions, 33001 Oviedo, Spain; 8 Department of Technology and Biotechnology, Instituto de Productos Lácteos de Asturias, Consejo Superior de Investigaciones Científicas (IPLA-CSIC), 33011 Oviedo, Spain; ladero@ipla.csic.es; 9 Grupo de Microbiología Molecular, Instituto de Investigación Sanitaria del Principado de Asturias (ISPA), 33011 Oviedo, Spain; 10 Department of Genetics, Faculty of Biology and Chemistry, University of Osnabrück, Barbarastrasse 11, D-49076 Osnabrück, Germany; jheinisc@uni-osnabrueck.de; 11 Departamento de Bioquímica y Biología Molecular, Universidad de Oviedo (UO), 33006 Oviedo, Spain

**Keywords:** monophasic variant of *Salmonella enterica* serovar Typhimurium, Spanish clone, ST19, multidrug resistance, resistance–virulence plasmids, IncC, plasmid evolution, phylogenetic analysis

## Abstract

Background/Objective: The present study focused on the analysis of the Spanish clone belonging to the successful 4,[5],12:i:- monophasic variant of *Salmonella enterica* serovar Typhimurium. Methods: All isolates of the clone recovered in a Spanish region from human clinical samples between 2008 and 2018 (N = 14) were investigated using microbiological approaches and genome sequence analysis. In addition, they were compared with isolates from the years 2000 to 2003 (N = 21), which were previously characterized but had not yet been sequenced. Results: Phylogenetic analyses indicate that all isolates are closely related (differing by 1 to 103 SNPs) but belong to two clades termed A and B. With few exceptions, clade A comprised isolates of the first period, also including two “older” control strains, LSP 389/97 and LSP 272/98. Clade B only contained isolates from the second period. Isolates from both periods were resistant to antibiotics and biocides, with almost all resistance genes located on large IncC plasmids, additionally carrying pSLT-derived virulence genes. The number of resistance genes was highly variable, resulting in a total of 22 ABR (antibiotic biocide resistance) profiles. The number of antibiotic resistance genes, but not that of biocide resistance genes, was considerably lower in isolates from the second than from the first period (with averages of 5.5 versus 9.6 genes). Importantly, IS*26*, which resides in multiple copies within these plasmids, appears to be playing a crucial role in the evolution of resistance, and it was also responsible for the monophasic phenotype, which was associated with four different deletions eliminating the *fljAB* region. Conclusions: the observed reduction in the number of antibiotic resistance genes could correlate with the loss of adaptive advantage originating from the ban on the use of antibiotics as feed additives implemented in the European Union since 2006, facilitated by the intrinsic instability of the IncC plasmids. Two consecutive IS*26* transposition events, which can explain both the clonal relationship of the isolates and their variability, may account for the observed *fljAB* deletions.

## 1. Introduction

The bacterial pathogen *Salmonella enterica* is one of the major causes of foodborne intestinal infections worldwide [1]. Although nearly 2600 serovars have been identified in this complex species [2], most cases of human salmonellosis worldwide can be attributed to just three of them, namely, *S*. Enteritidis, *S*. Typhimurium, and *S*. 4,[5],12:i:-. In fact, they accounted for 84.7% of all confirmed human infections in the European Union (EU) in 2022 [3]. Although *S*. 4,[5],12:i:- represents an independent serovar in terms of epidemiological surveillance [4], it actually is a monophasic variant of *S*. Typhimurium, which lacks the second-phase flagellar antigens (designated 1,2 in the antigenic formula of *S*. Typhimurium: 4,[5],12:i:1,2). This variant was responsible for 4.3% of the total cases of human salmonellosis confirmed in 2022 in the EU [5].

The 4,[5],12:i:- isolates investigated thus far can be assigned to four main clonal lines known as the Spanish, the European, the Southern European, and the United States clones [4,6,7]. These clones primarily differ both in their resistance towards antimicrobial drugs, with all except the U.S. clone being multidrug-resistant (MDR), and in the location of the encoding genes (residing either in specific chromosomal resistance regions or on plasmids of distinct incompatibility groups). They also differ by variable alterations affecting the *fljAB*-*hin* region, which encodes the second phase flagellin (*fljB* gene) and is responsible for phase variation in biphasic *S*. Typhimurium (governed by *hin* and *fljA*) [8]. Together with other characteristics of the monophasic clones, such as the sequence type (ST), predominant phage types, PFGE profiles, and phylogenetic relationships, this indicates that the monophasic clones originated from independent evolutionary events and do not necessarily derive from a common ancestor [9].

The Spanish monophasic clone emerged in Spain in the 1990s, recovered from pigs and pork products, and has been circulating mostly in the Iberian Peninsula [10,11,12,13,14,15,16,17]. Isolates of this clone show ST19 and were mainly associated with phage type U302, although DT193, DT208, and NT (not typeable with the available phage library) have also been reported [9,12,14]. The monophasic feature of the Spanish clone results from deletions within the *fljAB* region and its surrounding genes. Several deletions have already been identified, which were probably caused by transposition of the insertion sequence IS*26*. They all start at the same site within STM2758 (according to the *orf* nomenclature used for the annotation of the *S*. Typhimurium LT2 genome; accession number AE006468.1) but end at distinct positions [15,18,19,20].

The Spanish clone is typically resistant to ampicillin, chloramphenicol, gentamicin, streptomycin, sulphonamides, tetracyclines, and trimethoprim, although variations of the main phenotype have been detected [14,16,17]. The genes responsible for these resistances [*bla*_TEM-1B_, *cmlA1*, *aac(3)-IV*, *aadA1*, *aadA2*, *sul1*, *sul2*, *sul3*, *tet*(A), and *dfrA12*] are located on large plasmids (of ca. 150 to 200 kb) of the IncC (initially reported as IncA/C) incompatibility group which occasionally harbour the IncN replicon. Most of the resistance genes are supplied by elements involved in DNA mobility, including two class 1 integrons of the *sul1*-type, a class 1 integron of the *sul3*-type, and several transposons, specifically truncated versions of Tn*2*, Tn*21*, and Tn*1721*. One of the *sul1* integrons carries the *dfrA12*-*aadA2* genes in the variable region but lacks the 3′-conserved segment (CS) where the *sul1*-*qacE*Δ*1* genes reside; the other *sul1* integron does not contain gene cassettes in the variable region but retains an intact 3′-CS. On the other hand, the resistance genes *aadA2*, *cmlA1*, *aadA1,* and *qacH* are located within the *sul3* integron. The IncC plasmids of the Spanish monophasic clone also contain the *spv* operon (*spvRABCD*) along with other genes derived from pSLT, the virulence plasmid specific for *S*. Typhimurium [14,17]. pUO-STmRV1 (197,365 bp), the first resistance–virulence plasmid reported in the Spanish monophasic clone, was selected as a representative of the group and fully sequenced employing second- and third-generation technologies (i.e., Illumina and PacBio) [21].

To broaden our knowledge of the Spanish monophasic clone and determine its variability, all isolates of this clone recovered from human clinical samples in Asturias between 2008 and 2018 were sequenced with Illumina technology and experimentally characterized in the present study. In addition, the results were compared with those from clinical isolates obtained in the same region between the years 2000 and 2003, which were experimentally investigated in previous studies [14,15] but not sequenced until now. The obtained results were discussed in the context of the EU policies on the use of antibiotics and heavy metals in animal food production. It is worth noting that the province of Asturias in Northern Spain was the location where IncC plasmids of the Spanish monophasic clone were first identified [17].

## 2. Results

### 2.1. Incidence and General Properties of Monophasic Isolates of the Spanish Clone During the 2008–2018 Period

A total of 15 out of 615 monophasic isolates (2.4%) recovered from clinical samples in Asturias during 2008 to 2018 were assigned to the Spanish clone and initially selected for the present study (Table 1). The assignment was based on a resistance profile (phenotype and responsible genes) compatible with that typically associated with the Spanish clone or its variants, together with the presence of a plasmid of the IncC incompatibility group. Specifically, fourteen isolates were obtained from the faeces of different patients, and one was also present in the urine of one of those patients. For all fifteen isolates, the antigenic formula was experimentally determined to be 4,5,12:i:-.

### 2.2. Genome Sequence Analysis

The genomes of the 14 epidemiologically unrelated isolates were sequenced with short-read Illumina technology. Of the two isolates obtained from faeces and urine from the same patient, only the former was sequenced, as preliminary characterization indicated that both belonged to the same strain (Table 1). In addition, 21 clinical isolates from the 2000 to 2003 period and the old LSP 272/98 isolate [17] were also sequenced. The assembly size of the genomes ranged from 4,984,459 to 5,156,906 kb, built from 50 to 79 contigs (Table S1). All isolates could be assigned to ST19 by MLST performed in silico, as expected for the Spanish monophasic clone. Genome sequence analyses also led to the identification of genes related to both antibiotic and biocide resistance (Section 2.3). Moreover, the information gained for antibiotic resistance genes was consistent with the antibiogram results and with the presence of the relevant genes confirmed by PCR amplification, performed herein for the 2008–2018 isolates and previously reported for the older ones [14,17]. New information on the presence of biocide resistance genes and plasmid content was obtained by in silico analyses of all genome sequences.

The results from the plasmid analyses, as revealed by PLACNETw reconstruction, PBRT, PlasmidFinder, and pMLST, are summarized in Table 1. Apart from the IncC plasmid common to all isolates (Section 2.3), two of them (LSP 1142/03 and LSP 2/15) carried an IncI1-I(α) plasmid of ca. 96.4 and 116.2 kb, assigned to ST154 and ST259, respectively. In silico analysis of the genomes also showed the presence of a 54.8 kb plasmid in one isolate (LSP 54/18), whose incompatibility group could not be determined, and of smaller plasmids (ranging in size from 1.9 to 14.5 kb) in all isolates, except LSP 389/97. The latter plasmids were identified as OriColE, Col(BS512), Col8282, and Col(pHAD28) or had an unknown replicon. With regard to the phenotypes conferred by the identified plasmids, all were cryptic, except for the IncI1-I(α) plasmids, which contained the *strA*, *strB,* and *sul2* genes encoding resistance towards streptomycin and sulphonamides, and the IncC plasmids, which carried all other resistance genes found in the isolates, including antibiotic and biocide resistance genes, as well as virulence genes of pSLT.

### 2.3. Comparison of IncC Plasmids from the 2000 to 2003 and 2008 to 2018 Periods

The IncC plasmids of all sequenced clinical isolates were comprehensively characterized by means of bioinformatic analyses, and those belonging to older and more recent isolates were compared. The plasmids ranged in size between ca. 126 and 197.4 kb. The pMLST scheme for IncC plasmids includes the conserved *repA*, *parA*, *parB*, and *orf053* genes involved in plasmid replication and maintenance [22]. In silico pMLST performed for the IncC plasmids of the Spanish clone identified the repA_4*, parA_5*, parB_6, and A053_2 alleles, yielding an allelic profile different from those recorded for other plasmids of the group. The new ST was termed ST10* by the CGE tool since it was more closely related to ST10, differing by the alleles marked with an asterisk.

**Figure 1:**
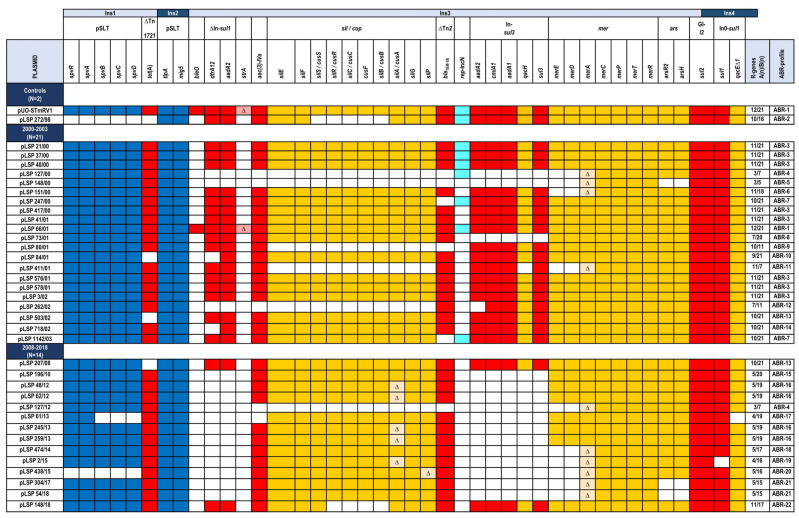
Resistance genes (antibiotics and biocides) and pSLT-derived virulence genes carried by IncC plasmids found in isolates of the Spanish monophasic clone of *Salmonella enterica* serovar Typhimurium. All plasmids are designated with a “p” followed by the code of the isolate where they were found, except for pUO-STmRV1, which would be pLSP 389/97 according to its origin. Virulence and resistance genes carried by these plasmids are shown in the order they appear in pUO-STmRV1, the fully sequenced plasmid of the control LSP 398/97 [21]. They are distributed in four regions (Ins1 to Ins4) inserted into the IncC backbone. In addition to these insertions, several genetic elements that supply virulence and resistance genes (namely, plasmid pSLT, transposons, integrons, and the GI-*sul2* genomic island), as well as the loci containing heavy metal resistance genes, are indicated at the upper part of the table. N, number of plasmids from each period. (n), number of antibiotic/biocide resistance genes; ABR, antibiotic and biocide resistance. Color code: dark blue, virulence genes; red, antibiotic resistance genes; orange, heavy metal resistance genes; pale blue, *rep* gene of the IncN incompatibility group; white: absent gene; ∆ on a paler tone, partially deleted gene. The Ins1 to Ins4 insertions are alternatively written on pale and dark blue backgrounds to highlight their positions.

As indicated before, IncC plasmids of the older isolates were previously characterized using a wide variety of experimental approaches [14,15,17], but they had not been previously sequenced. Sequence analysis performed herein provided additional information on virulence and resistance genes carried by them and allowed their comparison with those circulating in more recent years. Figure 1 shows the relevant genes in the order they appear in pUO-STmRV1, the plasmid of LSP 389/97, distributed along the four regions of foreign DNA (termed Ins1 to Ins4) inserted within the highly reduced backbone of the control plasmid [21].

**Figure 2:**
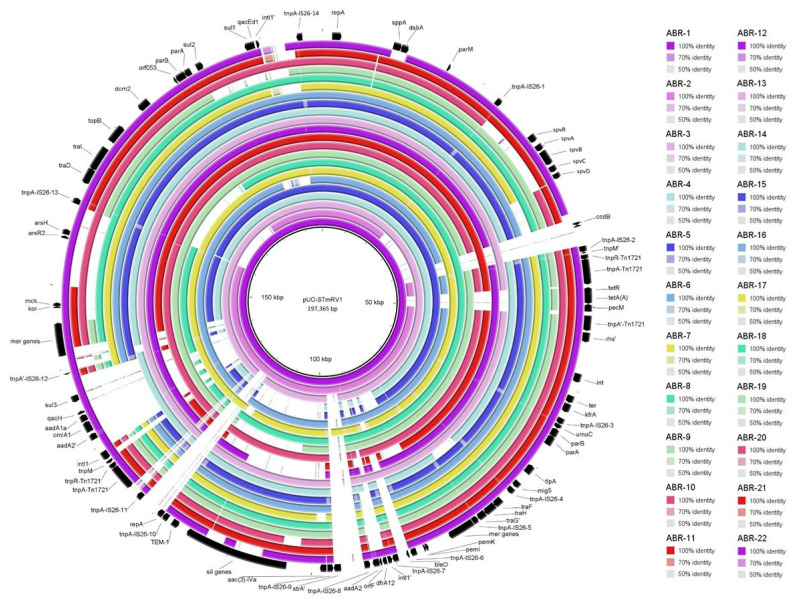
BRIG (Blast Ring Image Generator) comparison of IncC plasmids associated with different ABR (antibiotic biocide resistance) profiles found in clinical isolates of the Spanish monophasic clone of *Salmonella enterica* serovar Typhimurium. Each ring corresponds to a plasmid, according to the colour code specified on the right side of the figure. pUO-STmRV1 [21] was used as the reference (inner black ring). To generate the image, the concatenated contigs of the other plasmids, as identified by PLACNETw, were used. Relevant pUO-STmRV1 genes, represented by arrows indicating the direction of transcription, are displayed in the outer black ring. Note that only the presence/absence of genes is accurately represented with respect to the control but the figure does not necessarily reflect the synteny.

With regard to virulence genes, the *spv* locus (located within Ins1) was found to be intact in all plasmids, except pLSP 272/98 and pLSP 438/15, which lacked the entire locus, and pLSP 61/13, in which three out of the five *spv* genes were missing. This is in line with the original description of pLSP 272/98 as a resistance plasmid (formerly known as pUO-SR4 and pUO-STmR1), not as a resistance–virulence plasmid, based on the absence of the *spv* locus [14,17]. Yet, two other virulence genes of pSLT, *tlpA* and *mig5*, belonging to Ins2, were detected in all IncC plasmids under study, including pLSP 272/98. In relation to resistance profiles, the pUO-STmRV1-like plasmids of the Spanish monophasic clone displayed a great diversity (Figure 1). This is, for instance, reflected in the highly variable number of antibiotic resistance genes, which ranged between three and twelve, with only *sul2* shared by all plasmids. Variability was also observed for genes associated with resistance to heavy metals (mercury, silver, copper, and organo-arsenicals) and quaternary ammonium compounds. Taken together, resistances could be categorized into 22 ABR (antibiotic biocide resistance) gene profiles. A BRIG comparison of 22 plasmids selected as representatives of the different ABR profiles is shown in Figure 2.

Fourteen and ten profiles were associated with plasmids belonging to older and newer isolates, respectively, with only two of them (ABR4 and ABR13) being common to plasmids from both periods. The highest number of genes (33) was carried by the control pUO-STmRV1 and by pLSP 66/01, which shared the same profile (ABR-1) and were the only plasmids containing the *bleO* gene for bleomycin resistance. In contrast, the profile of pLSP 272/98 (ABR-2), also used as a control, was not shared by any other plasmid. The most frequent profile was ABR-3, which comprised all resistance genes present in pUO-STmRV1, except *bleO*, and was associated with eight plasmids, all found in isolates from the 2000 to 2003 period. The second most common profile was ABR-16, which was shared by four plasmids of recent isolates. The remaining profiles were associated either with one (fourteen profiles) or two (four profiles) isolates.

Variability concerning the antibiotic resistance mainly affected the class 1 integron of the *sul3* type and the defective class 1 integron of the *sul1* type with the 1600 bp/*dfrA12*-*aad2* variable region, which lacks the 3′-conserved segment where the *sul1* gene is located. In fact, both integrons were absent in all but two plasmids of the second period, as well as in two plasmids of the first period. Smaller deletions, which removed *dfrA12* or *aadA2*, appeared in the integrons of another three plasmids of the first period. Deletions affecting most other antibiotic resistance genes, including *tet*(A) and *bla*_TEM-1B_ (supplied by defective Tn*1721* and Tn*2* transposons, respectively) and *sul1* (carried by a second class 1 integron of the *sul1* type termed In0, which lacks the variable region), as well as *aac(3)-IVa* for gentamicin and tobramycin resistance, were also observed, though to a lesser extent. As indicated before, the only antibiotic resistance gene conserved in all plasmids appears to be *sul2,* which belongs to the GI*sul2* integrative element [23]. Similar to the antibiotic resistance loci, deletions that removed all or part of those associated with resistance to quaternary ammonium compounds and heavy metals, including copper, silver, mercury, and organo-arsenical compounds, were also detected.

### 2.4. Genetic Basis of the Monophasic Phenotype

**Figure 3:**
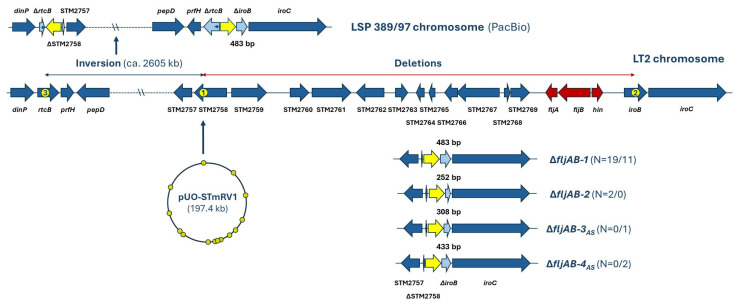
Deletions removing the *fljAB* genes in monophasic isolates of *Salmonella enterica* serovar Typhimurium belonging to the Spanish clone and recovered in Asturias during 2000–2003 and 2008–2018. Relevant genes on the *dinP* to *iroC* region of the chromosome of biphasic *S*. Typhimurium LT2 are shown for comparison (based on accession number AE006468.1; [24]). In these isolates, four different deletions (∆*fljAB-1*, ∆*fljAB-2*, ∆*fljAB-3_AS_*, and ∆*fljAB-4_AS_*) were identified as responsible for the monophasic phenotype, and a large inversion was also observed in the genome of LSP 389/97 sequenced with PacBio. The deletions, as well as the inversion, have probably been generated by three consecutive IS*26* transposition events (marked with 1, 2, and 3 in the LT2 chromosomal region; see Section 3 for details). Genes are represented by arrows pointing in the direction of transcription. Color code: dark blue arrows, intact genes; red arrows, genes encoding the second phase flagellin (*fljB*) and responsible for phase variation in biphasic *S*. Typhimurium (*hin* and *fljA*); pale blue arrows, truncated genes; yellow arrows and yellow circles, IS*26*, with 14 copies being present in pUO-STmRV1; small dark blue arrows, target site duplications (TDSs) generated upon transposition of IS*26* into *rtcB* (5′-GCATGGCG-3′ and 5′-CGCCATGC-3′; note that the position and orientation of one of them was altered due to the DNA inversion event). N, number of isolates from the 2000 to 2003/2008 to 2018 periods containing each of the indicated deletions, which differ in the length of the truncated *iroB* gene, as indicated in the figure. The truncated *iroB* found in the PacBio sequence of the LSP 389/97 chromosome corresponds to ∆*fljAB-1*.

To precisely determine the genetic basis of the monophasic phenotype, the *fljAB* chromosomal region of the isolates from the 2008 to 2018 period was compared with the corresponding region of Typhimurium LT2 (Figure 3).

All monophasic isolates had a large deletion, which starts at the same point within STM2758 (according to the annotation of the LT2 genome) and extends into *iroB* (STM2773), therefore removing the *fljA*, *fljB*, and *hin* genes. In all cases, only 100 bp out of the 1542 bp of the intact STM2758 was retained, while three different deletions, which eliminated 633, 808, and 683 bp out of 1116 bp of *iroB,* were identified. The observed deletions were designated as Δ*fljAB-1*, Δ*fljAB-3_AS_*, and Δ*fljAB-4_AS_*, following the numbering of previously published deletions for isolates from our region [15]. Δ*fljAB-1* was detected in eleven out of the fourteen isolates studied herein (78.6%), while Δ*fljAB-3_AS_* and Δ*fljAB-4_AS_*, which were newly discovered, were represented in only one and two isolates, respectively. The subscript AS (Asturias) was added to distinguish them from deletions with the same numbers previously identified in Spain outside Asturias [20]. Δ*fljAB-2*, reported in older isolates from our region [15], was not found in the more recent isolates. Yet, the same deletion was detected in other Spanish isolates, together with Δ*fljAB-3* and Δ*fljAB-*4, which are different from Δ*fljAB-3*_AS_ and Δ*fljAB-4*_AS_ [20]. Supporting previous findings, all isolates carried a copy of IS*26* between the start and end points of the observed deletions [15,20]. Interestingly, PacBio sequencing of the control isolate LSP 389/97 revealed a very large inversion encompassing ca. 2605 kb of chromosomal DNA, placed between truncated *rtcB* and STM2758 genes. This inversion was not present in the first analysis of this strain [15] and must have occurred at a later time during the cultivation in the laboratory (further elaborated on in Section 3).

### 2.5. Genomic Relationships Between the Isolates

**Figure 4:**
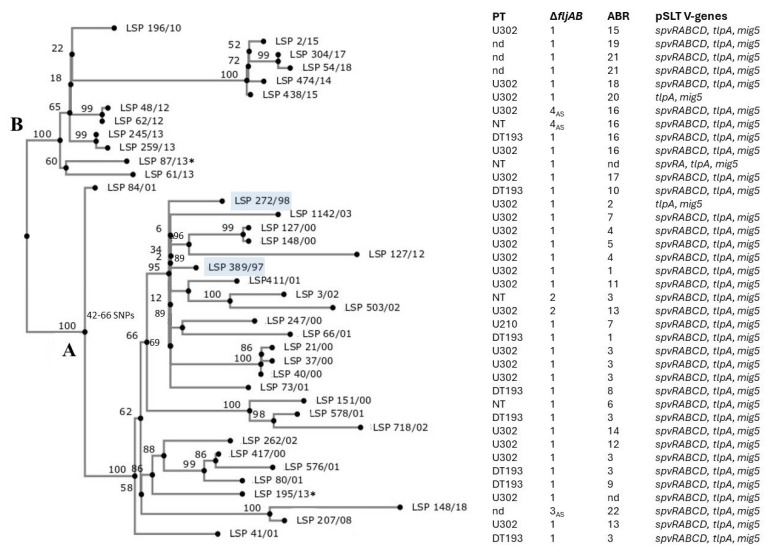
SNP-based phylogenetic tree showing the relationships between isolates of the Spanish monophasic clone. The tree was constructed with CSI Phylogeny 1.4 (https://cge.food.dtu.dk/services/CSIPhylogeny/; last accessed on 17 September 2021), using the genome of LSP 398/97 as a reference for SNP calling, together with another “old” isolate, LSP 272/98, both employed as controls throughout this work, and shown in blue boxes. Two food-derived isolates, not further discussed herein, are marked with an asterisk (*). Numbers at the nodes represent bootstrap values based on 1000 replicates. For all sequenced isolates, the phage type (PT), the type of *fljAB* deletion responsible for the monophasic phenotype, the antibiotic biocide resistance (ABR) profile, and the pSLT-derived virulence (V) genes carried by the IncC plasmids are shown. DT, Definitive Type; NT, not typeable; nd, not determined. For the control strains and the 2000–2003 isolates, information regarding phage type and the *fljAB* deletions has previously been reported [14,15,17]. The minimum and maximum numbers of SNPs ranged from 4 to 85 within clade A, from 1 to 57 SNPs within clade B, and up to a maximum of 103 between clades A and B.

To establish the genomic relationships between clinical isolates belonging to the Spanish clone, an SNP (single nucleotide polymorphism)-based phylogenetic tree was constructed, using 35 clinical isolates, 21 from the 2000 to 2003 period and 14 from the 2008 to 2018 period, as well as the control isolates LSP 389/97 and LSP 272/98. Two other isolates belonging to the Spanish monophasic clone and recovered from food samples in our region (LSP 87/13 and LSP 195/13) were also included in the tree. As shown in Figure 4, the 39 isolates were closely related, differing by a minimum of one (LSP 48/12 versus LSP 62/12) and a maximum of 103 (LSP 54/18 versus LSP 148/18) SNPs (Supplementary Table S2). Nevertheless, they formed two clades, termed A and B. Clade A comprised 27 isolates, varying by four to eighty-five SNPs, while the remaining twelve isolates clustered within clade B with one to fifty-seven SNPs. With two exceptions, clade A grouped isolates of the first period, including the “old” control strains LSP 389/97 and LSP 272/98, while clade B only contained isolates from the second period. To facilitate the categorization, Figure 4 also includes the phage type, the type of *fljAB* deletion, and the ABR profile and the *spv* genes contributed by the IncC plasmids.

Overall, 59.5% of the clinical isolates tested for phage type belonged to U302 (22/37, including LSP 389/97 and LSP 272/98). Others, like DT193 (8), U210 (1), and NT (4), which could not be typed with the phage library employed, were also detected (note that phage typing in Spain was discontinued in 2017). With regard to the *fljAB* deletions, Δ*fljAB-1* was the most common, shown by 86.5% of the clinical isolates (32/37), while Δ*fljAB-3_AS_* and Δ*fljAB-4_AS_* were only shown by a limited number of isolates from the second period (Section 2.4), and Δ*fljAB-2* was exclusively found in isolates from the first period [15]. Interestingly, the resistance profiles of the two isolates from the second period, which grouped in clade A (LSP 207/08 and LSP 148/18), comprised the highest number of antibiotic resistance genes (ABR-13 and ABR-22), with ten and eleven, respectively, versus three to five in the remaining profiles of isolates from the same period grouped into clade B.

## 3. Discussion

The present study focused on the incidence, variability, and evolution of the Spanish monophasic clone of *S*. Typhimurium. For this, all isolates of the clone recovered from human clinical samples between 2008 and 2018 in the northern Spanish region of Asturias were thoroughly characterized by conventional techniques combined with genome sequence analyses and compared with older isolates from the same region. During the 2008–2018 period, the Spanish clone accounted for 2.4% of the total monophasic isolates of clinical origin detected in Asturias. Other isolates were mostly assigned to the European ST34 clone, which is nowadays one of the most common foodborne pathogens involved in human infections worldwide [6]. In contrast, the Spanish clone belongs to ST19 and has a more restricted geographical distribution, being mainly detected in the Iberian Peninsula [10,11,12,13,14,15,16,17]. Moreover, its frequency has declined over time, from being the fourth most common serovar in 1998 in Spain [12]. The rise and decline of multidrug-resistant clones, which occur in successive waves, have determined the epidemiology of *S*. Typhimurium and its monophasic variant in Europe over the past decades [25,26,27]. Though the forces driving clone succession remain largely elusive, the early success of the Spanish clone and its subsequent replacement by the European clone in Spain have been proposed to be associated with changes in the European Union regulations for the use of antibiotics and heavy metals in food animal production [28]. In the present study, isolates from the second period were recovered soon after the implementation of the ban on the use of antibiotics as growth promoters for food animals, which became compulsory in 2006 [29]. This could explain the observed decrease in the number of antibiotic resistance genes, which dropped from an average of 9.6 during the first period, when the use of antibiotics as feed additives was still permitted, to an average of 5.5 during the second period. Compliance in Asturias with the EU regulations and Spanish legislation concerning the prudent use of antibiotics to combat resistance, both in veterinary and human medicine, has probably contributed to the observed reduction. Similarly, the widespread application of heavy metal compounds in food–animal production may have contributed to the selection and expansion of *S*. 4,[5],12:i:- isolates [28,30,31,32]. This could apply to the Spanish and the European clone since both contain genes conferring resistance to copper, silver, mercury, and arsenic compounds. However, while the MICs to mercury and arsenic were similar, MICs to silver and copper were much lower in the Spanish than in the European clone [28]. Mercury and arsenic compounds are increasingly regarded as undesirable contaminants in animal feed, with attempts to reduce their concentration and hence diminish the selective pressure for resistance mechanisms [33]. However, this does not concern copper and silver derivatives, which are still employed in food animal production. The increased resistance to these compounds due to the acquisition of SGI-4 by the European clone could explain the reduced appearance of the Spanish clone and its replacement by the European clone, which in Spain was estimated to occur around 2008 [28].

In the context of changing regulations on antibiotic and heavy metal usage in the EU, the structure of the IncC plasmids of the Spanish monophasic clone has a direct impact on the evolution of resistance. Thus, the presence of multiple copies of IS*26* (14 in pUO-STmRV1), together with many other potentially mobile genetic elements and repeated DNA [21], cause an intrinsic instability of these plasmids. This is consistent with the high number of variants identified herein and reported previously [14,16,17]. Interestingly, earlier attempts to cure the entire IncC plasmids from the Spanish clone failed but led to the identification of smaller variants originating from the loss of genes conferring resistance to ampicillin, chloramphenicol, gentamicin, streptomycin, tetracycline, and/or trimethoprim [16,17]. As shown in the present study, multiple variants lacking one or more of these and other resistance genes were detected in naturally occurring isolates infecting humans in our region. Accordingly, the IncC plasmids of the Spanish monophasic clone appear to combine a highly stable backbone (though largely degenerated [21]) with remarkably unstable accessory DNA. Together with efficient replication and partition mechanisms, vertical transmission of the plasmids is guaranteed by the toxin–antitoxin system encoded by the *ant*/*tox* (also known as *ata*/*tad*) genes within the IncC backbone, reinforced by the *ccdAB* genes of pSLT located within Ins1 [21]. As these plasmids are incapable of conjugative transfer due to extensive deletions within the *tra1* and *tra2* regions involved in conjugation, horizontal spread into non-clonal isolates does not occur [14,21]. Moreover, the accessory DNA is well-suited to respond to changes in selective pressure. In fact, the imprint of IS*26* on the evolution of pUO-STmRV1 was revealed by sequence analysis of the whole plasmid genome [21], and this insertion sequence may well be playing a major role in the generation of variants.

Apart from enabling readjustments in antimicrobial drug resistance, IS*26* is also responsible for the monophasic phenotype of the Spanish clone. Four different deletions were identified in our region (∆*fljAB*-1, ∆*fljAB*-2, ∆*fljAB*-3_AS_, and ∆*fljAB*-4_AS_), all removing the *fljAB* operon and its flanking DNA. The start point of these deletions within ST2758, common to all of them, and the variable end point located within the *iroB* gene, are separated by a single copy of IS*26*. This observation can be explained by two consecutive transposition events [34,35]. The first one would have been an intermolecular transposition of IS*26* into the chromosomal STM2758 gene of *S*. Typhimurium (marked with “1” in Figure 3). A pUO-STmRV1-like plasmid, already acquired by a biphasic isolate likely of phage type U302, probably acted as donor in this event. Although pU302L, found in *S*. Typhimurium U302 and carrying four copies of IS26, was suggested as the possible donor of the element [20], to the best of our knowledge this plasmid has not been detected in the Spanish clone. The second event would have been an intramolecular transposition of IS*26* from ST2758 into *iroB* (“2” in Figure 3) using the cis-pathway. This would remove the intervening DNA leading to the monophasic phenotype, and it must have occurred independently more than once to account for the different deletions observed. A similar event would explain deletions extending beyond *iroB*, which were reported in Spain by other authors [18,20]. Of note, after acquiring the Δ*fljAB-1* deletion, IS*26* transposition into the *rtcB* gene (“3” in Figure 3) of the control LSP 389/97 isolate using the trans-pathway led to the inversion of a large DNA segment, spanning between ∆STM2758 and Δ*rtcB*. This view is substantiated by the fact that TSDs (target site duplications) of 8 bp, originally present in *rtcB*, were observed, although the position and orientation of one of the repeats changed upon inversion (5′-GCATGGCG-3′ and 5′-CGCCATGC-3′). Since this inversion did not yet occur when LSP 389/98 was first tested for the *fljAB* deletion [15], this is a good example of the “hyperactivity” of IS*26*, which once incorporated into a new DNA molecule will continue to act as an efficient motor for further evolution.

Apart from explaining the monophasic phenotype, the proposed scenario is consistent with the close relationship existing between the monophasic isolates of the Spanish clone, as previously revealed by traditional typing techniques, like phage typing, PFGE, MLVA, and microarray analysis [14,15] and corroborated by SNP-based phylogenetic analysis performed herein. In fact, all isolates of the Spanish clone may derive from a single biphasic ancestor of *S*. Typhimurium U302 that either acquired pUO-STmRV1 or in which the resistance–virulence plasmid was first assembled. After transposition of IS*26* from the plasmid into STM2758, intra-clonal evolution would not only explain the various deletions leading to the monophasic phenotype but also the differences in phage type, resistance profiles, and the limited number of SNPs detected within the genomes.

## 4. Materials and Methods

### 4.1. Isolate Selection and Preliminary Characterization

All *S*. 4,[5],12:i:- isolates recovered from human clinical samples in Asturias (a Northern Spanish region) during the 2008–2018 period (615) were experimentally tested for antibiotic resistance properties, resistance genes, and plasmid content. Fifteen of them, displaying properties consistent with the Spanish clone, were selected for the present study (Table 1). They were obtained from the faeces of different patients with gastroenteritis at four hospitals in the region, and also from the urine of one of the patients. For all of them, the serotype was experimentally determined, and for those recovered before 2017, the phage type was also established. The information was provided by the Laboratory of Public Health (LSP) of Asturias. Antibiotic susceptibility testing and detection of the resistance genes were experimentally performed as in [36]. Screening for the IncC and IncN replicons was carried out by the PBRT (PCR-based replicon typing) method [37]. LSP 389/97 (the isolate carrying pUO-STmRV1), LSP 272/98, which are two of the oldest monophasic isolates of the Spanish clone recovered in our region [17], and *S*. Typhimurium LT2, the type strain of the genus *Salmonella* [24], were used as controls in different experiments.

### 4.2. Whole Genome Sequencing and Bioinformatics Analysis

Fourteen epidemiologically unrelated isolates belonging to the Spanish clone and recovered from clinical samples during the 2008–2018 period were sequenced using short-read Illumina technology, as described [36]. Another 21 isolates of the same clone, obtained between 2000 and 2003 and characterized in previous studies [14,15], as well as LSP 272/98, were additionally sequenced. The obtained raw reads were assembled into contigs with the VelvetOptimiser.pl script implemented in the online version of PLACNETw (https://castillo.dicom.unican.es/upload/; last accessed on 12 April 2021). The assembled genomes were annotated by the NCBI Prokaryotic Genome Annotation Pipeline (PGAP; https://www.ncbi.nlm.nih.gov/genome/annotation_prok/; last accessed on 28 June 2021) and deposited in GenBank under the accession numbers provided in Table S1. Bioinformatic analyses were performed both with PLACNETw and several tools available at the Center for Genomic Epidemiology (CGE; https://www.genomicepidemiology.org/services/; last accessed on 12 June 2025) of the Technical University of Denmark (DTU), including SeqSero, MLST, ResFinder, PlasmidFinder, and pMLST. Heavy metal resistance genes were identified by MEGares 2.0 and with a customized database containing heavy metal resistance genes previously detected in *S. enterica*, which was screened with MyDbFinder (CGE, DTU). Analysis of *fljAB* regions was performed with the aid of BLASTn and CLONE Manager (ClonSuit9), and also with a second in-house customized database comprising all open reading frames located between STM2752 and the *iroC* gene in the chromosome of biphasic *S*. Typhimurium LT2 (accession number AE006468.1). The entire sequence of LSP 389/97, including the chromosome (accession no. CP018219) and plasmid pUO-STmRV1 (accession no. CP018220), has been previously generated by combining short-read Illumina and long-read PacBio technologies [21]. Both sequences were used as controls for the in silico analyses. Graphic representations derived from IncC plasmids were generated with BRIG (Blast Ring Image Generator; https://sourceforge.net/projects/brig/; last accessed on 10 July 2025), using pUO-STmRV1 as the reference.

### 4.3. Phylogenetic Analysis

The phylogenetic relationships between isolates of the Spanish monophasic clone sequenced in the present study (a total of 36) were inferred with the CSI tool (version 1.4) available at the CGE website (last accessed on 17 September 2021). LSP 389/97 and two isolates of the same clone recovered from food samples in our region (LSP 87/13 and LSP 195/13) were also included in the tree. The pipeline was run with default parameters, using the genome of LSP 389/97 as reference for SNP calling, and the resulting SNP matrix is shown in Table S2. Bootstrap support for the consensus phylogenetic tree was based on 1000 replicates [38].

## Figures and Tables

**Table 1:** Origin, antimicrobial resistance properties, and plasmid content of clinical isolates belonging to the Spanish monophasic clone of *Salmonella enterica* serovar Typhimurium.

Isolate ^a^	Patient ^b^ Sex/Age Hospital ^c^	AR ^d^ Phenotype Encoded by Resistance Plasmids	R-Plasmid (Inc; Size in bp) ^e^ Other Plasmids (Size in bp) ^f^
LSP 389/97	F/17 HUSA	AMP, AMC, CHL, GEN, TOB, STR, SUL, TET, TMP	**IncC/**IncN (pUO-STmRV1; 197,365) *
LSP 272/98	M/59 HJ	AMP, AMC, CHL, GEN, TOB, STR, SUL, TMP	**IncC/**IncN **(137,711)** unk * (4073), Col(BS512) * (2101)
LSP 21/00	F/1 HCN	AMP, AMC, CHL, GEN, TOB, STR, SUL, TET, TMP	**IncC**/IncN (**166,540**) unk * (4073), Col(BS512) * (2101)
LSP 37/00	F/1 HCN	AMP, AMC, CHL, GEN, TOB, STR, SUL, TET, TMP	**IncC**/IncN (**167,805**) unk * (4073), Col(BS512) * (2101)
LSP 40/00	unk/unk unk	AMP, AMC, CHL, GEN, TOB, STR, SUL, TET, TMP	**IncC**/IncN (**166,539**) unk * (4073), Col(BS512) * (2101)
LSP 127/00	F/1 HUCA	SUL, TET	**IncC**/IncN (**129,018**) unk * (4073), Col(BS512) * (2101)
LSP 148/00	F/2 HUC	SUL, TET	**IncC** (**129,267**) Col(BS512) * (2101)
LSP 151/00	M/2 HUC	AMP, AMC, CHL, GEN, TOB, STR, SUL, TET, TMP	**IncC** (**156,873**) unk * (4073), unk * (3373), Col(BS512) * (2101), OriColE * (1888)
LSP 247/00	M/11 HUC	CHL, GEN, TOB, STR, SUL, TET, TMP	**IncC** (**173,621**) unk * (4073), Col(BS512) * (2101)
LSP 417/00	F/unk HUC	AMP, AMC, CHL, GEN, TOB, STR, SUL, TET, TMP	**IncC** (**168,460**) unk (14,418), unk * (4074), Col(BS512) * (2101)
LSP 41/01	F/55 HCN	AMP, AMC, CHL, GEN, TOB, STR, SUL, TET, TMP	**IncC** (**168,287**) unk (7940), Col(BS512) * (2101)
LSP 66/01	F/4 HCN	AMP, AMC, CHL, GEN, TOB, STR, SUL, TET, TMP	**IncC** (**176,490**) unk * (4073), Col(BS512) * (2101)
LSP 73/01	M/4 HJ	AMP, AMC, GEN, TOB, STR, SUL, TET, TMP	**IncC** (**147,514**) unk * (4073), Col(BS512) * (2101)
LSP 80/01	F/1 HUC	CHL, GEN, TOB, STR, SUL, TET, TMP	**IncC** (**167,501**) unk * (4074), Col(BS512) * (2101)
LSP 84/01	M/unk HUC	AMP, AMC, CHL, GEN, TOB, STR, SUL	**IncC** (**163,450**) unk (10,648), Col(BS512) * (2101)
LSP 411/01	M/unk HCN	AMP, AMC, CHL, GEN, TOB, STR, SUL, TET, TMP	**IncC** (**125,916**) unk * (4073), Col(BS512) * (2101)
LSP 576/01	M/unk HUC	AMP, AMC, CHL, GEN, TOB, STR, SUL, TET, TMP	**IncC** (**168,372**) unk (14,418), unk * (4096), Col(BS512) * (2101)
LSP 578/01	F/3 HUC	AMP, AMC, CHL, GEN, TOB, STR, SUL, TET, TMP	**IncC** (**161,336**) unk * (3373), Col(BS512) * (2101), OriColE * (1888)
LSP 3/02	M/1 HUCA	AMP, AMC, CHL, GEN, TOB, STR, SUL, TET, TMP	**IncC** (**173,402**) unk * (4073), Col(BS512) * (2101), unk * (1989), OriColE * (1888)
LSP 262/02	M/28 HUC	AMP, AMC, CHL, STR, SUL, TET	**IncC** (**165,469**) unk * (4074), Col(BS512) * (2101)
LSP 503/02	F/30 HUCA	AMP, AMC, CHL, GEN, TOB, STR, SUL, TMP	**IncC** (**169,183**) unk * (4073), Col(BS512) * (2101), OriColE * (1888)
LSP 718/02	F/5 HUCA	AMP, AMC, CHL, GEN, TOB, STR, SUL, TET	**IncC** (**159,273**) unk * (4073), unk * (3373), Col(BS512) * (2101), OriColE * (1888)
LSP 1142/03	F/73 HUC	CHL, GEN, TOB, STR, SUL, TET, TMP STR, SUL	**IncC**/IncN (**183,291**) **IncI1-I(α)/ST154-(96,419)** unk * (4073), Col(BS512) * (2101)
LSP 207/08	F/70 HMN	AMP, AMC, CHL, GEN, TOB, STR, SUL, TMP	**IncC (164,679)** unk (14,518), Col(BS512) + Col8282 (12,901), unk (5052)
LSP 196/10	F/32 HJ	AMP, AMC, GEN, TOB, SUL, TET	**IncC (155,741)** unk * (4073), Col(BS512) * (2101), oriColE * (1888)
LSP 48/12	F/11 HUCA	AMP, AMC, GEN, TOB, SUL, TET	**IncC (157,533)** unk (4594), Col(BS512) (3142), unk (2677)
LSP 62/12	F/57 HUC	AMP, AMC, GEN, TOB, SUL, TET	**IncC (152,856)** Col(BS512) (8718), oriColE (1044)
LSP 127/12	M/73 HUC	SUL, TET	**IncC (128,459)** Col(pHAD28) * (4418), oriColE * (4230), unk (4203), oriColE * (3830), Col(BS512) (3143)
LSP 61/13	M/4 HUC	AMP, AMC, SUL, TET	**IncC (150,970)** Col(BS512) (3143), oriColE (2570)
LSP 245/13	F/25 HJ	AMP, AMC, GEN, TOB, SUL, TET	I**ncC (143,284)** unk (14,518), unk (5424), Col(BS512) (3652), unk (2677)
LSP 259/13	F/54 HJ	AMP, AMC, GEN, TOB, SUL, TET	**IncC (157,802)** unk (5115), Col(BS512) (3651), unk (2677)
LSP 474/14	M/56 HUSA	AMP, AMC, GEN, TOB, SUL, TET	**IncC (155,767)** unk (7281), unk * (4073), unk (2622)
LSP 2/15	M/75 HUSA	AMP, AMC, GEN, TOB, SUL, TET STR, SUL	**IncC (131,910)** **IncI1-I(α)/ST259 (116,151)** unk (4203), unk (3924), oriColE (2667)
LSP 438/15	M/52 HUCA	AMP, AMC, GEN, TOB, SUL, TET	**IncC (141,765)** unk (4203), unk (2677), Col(BS512) (2622)
LSP 304/17	F/6 HUSA	AMP, AMC, GEN, TOB, SUL, TET	**IncC (140,638)** Col8282 * (4091), unk * (4073), Col(BS512) (3142), oriColE (2677)
LSP 54/18 (LSP 53/18)	F/70 HUC	AMP, AMC, GEN, TOB, SUL, TET	**IncC (137,672)** unk * (54,805), Col(BS512) (4221), unk * (4091), Col8282 (3142), oriColE (2677)
LSP 148/18	F/unk HUSA	AMP, AMC, CHL, GEN, TOB, STR, SUL, TET, TMP	**IncC-(158,125)** unk (8840), Col(BS512) (2621)

^a^, LSP, “Laboratorio de Salud Pública” (Asturias, Spain). All isolates shared the antigenic formula 4,5,12:i:- and the sequence type ST19. LSP389/97, the oldest isolate of the Spanish clone detected in our region, and LSP 272/98 (both underlined) were included for comparison [17]. Each isolate was obtained from faeces of a different patient, except LSP 53/18 (shown in parentheses), which was recovered from the urine of the same patient as LSP 54/18. The two isolates shared identical resistance properties and were considered as a single strain. ^b^, M, male; F, female; unk, unknown. ^c^, HUSA, “Hospital Universitario San Agustín”; HJ, “Hospital de Jarrio”; HCN, “Hospital Cangas del Narcea”; HUCA, “Hospital Universitario Central de Asturias”; HUC, “Hospital Universitario de Cabueñes”; HMN, “Hospital Monte Naranco”; unk, unknown. ^d^, AR, antibiotic resistance; AMP, ampicillin; AMC, amoxicillin–clavulanic acid; CHL, chloramphenicol; GEN, gentamicin; TOB; tobramycin; STR, streptomycin; SUL, sulphonamides; TET, tetracycline; TMP, trimethoprim. ^e^, Inc, incompatibility group, with resistance (R-) plasmids shown in bold; ^f^, unk, unidentified replicon; *, plasmid assembled in one contig and circularized in silico. For the control strains and the 2000–2003 isolates, information regarding antibiotic resistance encoded by plasmids was previously published [14,17].

## Data Availability

The genome sequences generated in the present study were deposited in the GenBank database under BioProject PRJNA742063, under the accession numbers compiled in Table S1.

## Supplementary Materials

The following supporting information can be downloaded at https://www.mdpi.com/article/10.3390/antibiotics14070711/s1, Table S1: Accession numbers of the genomes of clinical isolates belonging to the Spanish monophasic clone of *Salmonella enterica* sequenced in this study. Parameters related to the quality of the assemblies. Table S2: Pairwise distance matrix calculated from SNPs in the genomes of isolates belonging to the Spanish monophasic clone of *Salmonella enterica*.
