# Supplementary material for: Epidemiological Surveillance, Variability, and Evolution of Isolates Belonging to the Spanish Clone of the 4,[5],12:i:- Monophasic Variant of Salmonella enterica Serovar Typhimurium
Source: Antibiotics (Basel). 2025 Jul 16;14(7):711. doi: 10.3390/antibiotics14070711 (PMC12291984; doi:10.3390/antibiotics14070711)
Supplement: Supplementary file 1 [file antibiotics-14-00711-s001.zip › antibiotics-3740716-supplementary.pdf]

antibiotics

MDPI

Supplementary material

Epidemiological surveillance and genomic analysis of isolates belonging to the Spanish clone of the monophasic 4,[5],12:i:- variant of *Salmonella enterica* serovar Typhimurium

Table S1. Accession numbers of the genomes of clinical isolates belonging to the Spanish monophasic clone of *Salmonella enterica* sequenced in this study. Parameters related to the quality of the assemblies

Isolate[a]	kmer	Contigs	N50	Longest contig (bp)	Total bp in contigs	Contigs > 1 kb	Library	Coverage	GenBank accession number
LSP 272/98	71	163	222788	600312	5007057	74	467 +/- 19	41x	JAHTGW000000000
LSP 21/00	75	134	222800	600267	5000909	60	465 +/- 17	32x	JAHTGY000000000
LSP 37/00	89	123	293481	746199	5046701	66	512+/-132	30x	JAHTGZ000000000
LSP 40/00	91	111	225736	746224	50466787	66	500+/-133	27x	JAHTHA000000000
LSP 127/00	89	126	225732	746333	5044571	69	493+/-129	28x	JAHTGX000000000
LSP 148/00	89	104	225732	746309	5009823	57	490+/-131	30x	JAHTHB000000000
LSP 151/00	89	119	225732	746181	5030031	66	495+/-132	24x	JAHTHC000000000
LSP 247/00	89	128	225732	746065	5058416	68	496+/-130	25x	JAHTHD000000000
LSP 417/00	91	120	293448	746314	5064494	70	510+/-136	26x	JAHTHE000000000
LSP 41/01	91	121	225736	746272	5053648	69	507+/-134	31x	JAHTHF000000000
LSP 66/01	91	116	225736	746102	5058931	66	523+/-136	23x	JAHTHG000000000
LSP 73/01	91	110	225736	746267	5027708	60	489+/-127	32x	JAHTHH000000000
LSP 80/01	93	119	225740	746393	5049561	67	490+/-128	20x	JAHTHI000000000
LSP 84/01	89	120	225732	746252	5048827	63	477+/-128	24x	JAHTHJ000000000
LSP 411/01	91	91	225736	746305	5004997	53	473+/-125	25x	JAHTHK000000000
LSP 576/01	91	122	225736	746185	5040210	69	465+/-127	23x	JAHTHL000000000

Isolate[a]	kmer	Contigs	N50	Longest contig (bp)	Total bp in contigs	Contigs > 1 kb	Library	Coverage	GenBank accession number
LSP 578/01	89	120	225732	746181	5042057	67	457+/-125	30x	JAHTHM000000000
LSP 3/02	91	124	225736	745992	5055446	71	478+/-135	25x	JAHTHN000000000
LSP 262/02	91	113	225736	766101	5045253	68	467+/-131	29x	JAHTHO000000000
LSP 503/02	91	106	225736	746084	5022648	62	514+/-132	30x	JAHTHP000000000
LSP 718/02	89	112	222489	600325	5045110	58	510+/- 132	27x	JAHTHQ000000000
LSP 1142/03	93	139	293478	746304	5131838	76	523+/-139	31x	JAHTHR000000000
LSP 207/08	131	144	223629	600357	5069991	61	469 +/- 59	24x	JAHTHS000000000
LSP 196/10	93	88	362876	746223	4985176	52	518+/-142	40x	JAHTHT000000000
LSP 48/12	131	101	362946	746782	4985454	50	428 +/- 130	48x	JAHTHU000000000
LSP 62/12	131	96	363024	746776	4984459	50	411 +/- 128	47x	JAHTHV000000000
LSP 127/12	131	110	223364	630318	5049893	51	308 +/- 101	76x	JAHTHW000000000
LSP 61/13	131	127	223423	600379	5031963	57	341 +/- 91	59x	JAHTHX000000000
LSP 245/13	131	116	223390	523102	4987608	50	324 +/- 103	70x	JAHTIA000000000
LSP 259/13	131	113	225816	600373	4987131	50	326 +/- 97	71x	JAHTIB000000000
LSP 474/14	131	123	223412	547024	5040436	60	382 +/- 116	59x	JAHTIC000000000
LSP 2/15	131	182	223391	600379	5156906	79	273 +/-89	48x	JAHTID000000000
LSP 438/15	131	118	225816	746536	5022939	54	411 +/- 126	46x	JAHTIE000000000
LSP 304/17	131	126	223404	547082	5029841	61	338 +/- 106	72x	JAHTIF000000000
LSP 54/18	131	174	222117	600379	5118629	78	388 +/- 125	57x	JAHTIG000000000
LSP 148/18	131	120	222112	521454	5046163	60	404 +/- 125	58x	JAHTIH000000000

[a], LSP, "Laboratorio de Salud Pública"(Asturias, Spain)

Table S2. Pairwise distance matrix calculated from SNP in the genomes of isolates belonging to the Spanish monophasic clone of *Salmonella enterica*.

	LSP 1142/03	LSP 127/00	LSP 127/12	LSP 148/00	LSP 148/18	LSP 151/00	LSP 195/13	LSP 196/10	LSP 207/08	LSP 21/00	LSP 2/15	LSP 245/13	LSP 247/00	LSP 259/13	LSP 262/02	LSP 272/98	LSP 3/02	LSP 304/17	LSP 37/00	LSP 389/97	LSP 40/00	LSP 41/01	LSP 411/01	LSP 417/00	LSP 438/15	LSP 474/14	LSP 48/12	LSP 503/02	LSP 54/18	LSP 576/01	LSP 578/01	LSP 61/13	LSP 62/12	LSP 66/01	LSP 718/02	LSP 73/01	LSP 80/01	LSP 84/01	LSP 87/13
**LSP 1142/03**	0	45	57	39	74	59	53	71	57	44	86	69	40	71	49	45	46	89	45	31	42	45	34	42	85	87	71	53	91	54	54	67	70	44	61	40	49	44	66
**LSP 127/00**	45	0	50	12	73	60	48	64	56	45	85	62	41	64	50	36	49	88	40	30	45	44	35	45	84	86	64	54	90	53	53	66	63	45	62	39	50	45	67
**LSP 127/12**	57	50	0	44	79	72	60	80	62	57	91	76	49	78	64	54	57	94	56	36	55	58	49	57	90	92	78	64	96	69	69	74	77	57	76	55	64	59	81
**LSP 148/00**	39	12	44	0	67	54	44	68	50	41	79	64	35	66	46	40	43	82	38	24	41	40	33	41	78	80	66	48	84	53	51	60	65	39	56	35	46	43	61
**LSP 148/18**	74	73	79	67	0	81	67	87	27	74	98	83	66	85	71	71	74	101	73	53	72	65	66	64	97	99	85	81	103	76	78	81	84	74	85	72	71	66	88
**LSP 151/00**	59	60	72	54	81	0	58	80	64	59	93	76	53	78	52	62	59	96	58	46	55	54	51	51	92	94	78	68	98	61	27	74	77	57	38	55	54	45	73
**LSP 195/13**	53	48	60	44	67	58	0	66	50	47	79	62	45	64	40	48	51	82	46	34	47	42	43	35	78	80	64	56	84	47	51	60	63	49	60	47	40	41	61
**LSP 196/10**	71	64	80	68	87	80	66	0	70	71	49	14	65	16	70	62	71	52	72	54	69	62	61	61	48	50	16	80	54	71	75	34	15	75	84	71	68	45	39
**LSP 207/08**	57	56	62	50	27	64	50	70	0	57	81	66	49	68	54	54	57	84	56	36	55	48	49	47	80	82	68	64	86	59	61	64	67	57	68	55	54	49	71
**LSP 21/00**	44	45	57	41	74	59	47	71	57	0	86	67	38	69	47	41	42	89	9	31	4	47	36	44	85	87	69	51	91	56	50	69	68	42	61	42	49	42	66
**LSP 2/15**	86	85	91	79	98	93	79	49	81	86	0	45	78	47	83	83	86	9	85	65	84	75	78	76	5	7	47	93	11	88	90	45	46	86	97	84	83	60	52
**LSP 245/13**	69	62	76	64	83	76	62	14	66	67	45	0	61	2	66	58	67	48	68	50	65	60	59	59	44	46	12	76	50	69	71	30	11	71	80	69	66	41	37
**LSP 247/00**	40	41	49	35	66	53	45	65	49	38	78	61	0	63	45	39	38	81	39	23	34	43	32	38	77	79	63	45	83	52	50	61	62	36	57	38	45	38	64
**LSP 259/13**	71	64	78	66	85	78	64	16	68	69	47	2	63	0	68	60	69	50	70	52	67	62	61	61	46	48	14	78	52	71	73	32	13	73	82	71	68	43	39
**LSP 262/02**	49	50	64	46	71	52	40	70	54	47	83	66	45	68	0	48	49	86	46	38	47	42	43	29	82	84	68	58	88	39	45	64	67	47	56	43	30	33	61
**LSP 272/98**	45	36	54	40	71	62	48	62	54	41	83	58	39	60	48	0	43	86	40	28	41	46	35	47	82	84	60	54	88	55	51	66	59	45	60	41	52	43	69
**LSP 3/02**	46	49	57	43	74	59	51	71	57	42	86	67	38	69	49	43	0	89	45	31	40	49	36	46	85	87	69	37	91	60	54	69	68	44	63	44	51	44	70
**LSP 304/17**	89	88	94	82	101	96	82	52	84	89	9	48	81	50	86	86	89	0	88	68	87	78	81	79	6	8	50	96	2	91	93	48	49	89	100	87	86	63	55
**LSP 37/00**	45	40	56	38	73	58	46	72	56	9	85	68	39	70	46	40	45	88	0	30	9	46	35	45	84	86	70	52	90	55	49	66	69	41	58	37	50	41	65
**LSP 389/97**	31	30	36	24	53	46	34	54	36	31	65	50	23	52	38	28	31	68	30	0	29	32	23	31	64	66	52	38	70	43	43	48	51	31	50	29	38	33	55
**LSP 40/00**	42	45	55	41	72	55	47	69	55	4	84	65	34	67	47	41	40	87	9	29	0	47	34	42	83	85	67	49	89	54	50	67	66	42	61	42	49	38	66
**LSP 41/01**	45	44	58	40	65	54	42	62	48	47	75	60	43	62	42	46	49	78	46	32	47	0	39	35	74	76	62	56	80	47	49	56	61	45	58	43	40	37	57
**LSP 411/01**	34	35	49	33	66	51	43	61	49	36	78	59	32	61	43	35	36	81	35	23	34	39	0	36	77	79	61	41	83	44	46	59	60	38	55	34	43	36	60
**LSP 417/00**	42	45	57	41	64	51	35	61	47	44	76	59	38	61	29	47	46	79	45	31	42	35	36	0	75	77	61	51	81	16	46	57	60	44	55	42	13	34	58
**LSP 438/15**	85	84	90	78	97	92	78	48	80	85	5	44	77	46	82	82	85	6	84	64	83	74	77	75	0	4	46	92	8	87	89	44	45	85	96	83	82	59	51

**LSP 474/14**	87	86	92	80	99	94	80	50	82	87	7	46	79	48	84	84	87	8	86	66	85	76	79	77	4	0	48	94	10	89	91	46	47	87	98	85	84	61	53
**LSP 48/12**	71	64	78	66	85	78	64	16	68	69	47	12	63	14	68	60	69	50	70	52	67	62	61	61	46	48	0	78	52	71	73	32	1	73	82	71	68	43	39
**LSP 503/02**	53	54	64	48	81	68	56	80	64	51	93	76	45	78	58	54	37	96	52	38	49	56	41	51	92	94	78	0	98	65	63	74	77	53	70	51	58	53	75
**LSP 54/18**	91	90	96	84	103	98	84	54	86	91	11	50	83	52	88	88	91	2	90	70	89	80	83	81	8	10	52	98	0	93	95	50	51	91	102	89	88	65	57
**LSP 576/01**	54	53	69	53	76	61	47	71	59	56	88	69	52	71	39	55	60	91	55	43	54	47	44	16	87	89	71	65	93	0	56	69	70	58	67	52	25	42	70
**LSP 578/01**	54	53	69	51	78	27	51	75	61	50	90	71	50	73	45	51	54	93	49	43	50	49	46	46	89	91	73	63	95	56	0	71	72	52	23	50	49	40	70
**LSP 61/13**	67	66	74	60	81	74	60	34	64	69	45	30	61	32	64	66	69	48	66	48	67	56	59	57	44	46	32	74	50	69	71	0	31	67	78	65	64	41	31
**LSP 62/12**	70	63	77	65	84	77	63	15	67	68	46	11	62	13	67	59	68	49	69	51	66	61	60	60	45	47	1	77	51	70	72	31	0	72	81	70	67	42	38
**LSP 66/01**	44	45	57	39	74	57	49	75	57	42	86	71	36	73	47	45	44	89	41	31	42	45	38	44	85	87	73	53	91	58	52	67	72	0	61	40	49	44	66
**LSP 718/02**	61	62	76	56	85	38	60	84	68	61	97	80	57	82	56	60	63	100	58	50	61	58	55	55	96	98	82	70	102	67	23	78	81	61	0	55	60	53	75
**LSP 73/01**	40	39	55	35	72	55	47	71	55	42	84	69	38	71	43	41	44	87	37	29	42	43	34	42	83	85	71	51	89	52	50	65	70	40	55	0	45	40	58
**LSP 80/01**	49	50	64	46	71	54	40	68	54	49	83	66	45	68	30	52	51	86	50	38	49	40	43	13	82	84	68	58	88	25	49	64	67	49	60	45	0	37	61
**LSP 84/01**	44	45	59	43	66	45	41	45	49	42	60	41	38	43	33	43	44	63	41	33	38	37	36	34	59	61	43	53	65	42	40	41	42	44	53	40	37	0	40
**LSP 87/13**	66	67	81	61	88	73	61	39	71	66	52	37	64	39	61	69	70	55	65	55	66	57	60	58	51	53	39	75	57	70	70	31	38	66	75	58	61	40	0
